# Reply to ‘Antipsychotics with similar association kinetics at dopamine D_2_ receptors differ in extrapyramidal side-effects’

**DOI:** 10.1038/s41467-018-05678-4

**Published:** 2018-09-03

**Authors:** David A. Sykes, J. Robert Lane, Monika Szabo, Ben Capuano, Jonathan A. Javitch, Steven J. Charlton

**Affiliations:** 1 School of Life Sciences, Queen’s Medical Centre, University of Nottingham, Nottingham, NG7 2UH UK; 2 Centre of Membrane and Protein and Receptors (COMPARE), University of Birmingham and University of Nottingham, Midlands, UK; 3 Drug Discovery Biology, Monash Institute of Pharmaceutical Sciences, Monash University, 381 Royal Parade, Parkville, VIC 3052 Australia; 4 Medicinal Chemistry, Monash Institute of Pharmaceutical Sciences, Monash University, 381 Royal Parade, Parkville, VIC 3052 Australia; 5 Department of Psychiatry, Columbia University, New York, NY 10032 USA; 6 Integrative Neuroscience, New York State Psychiatric Institute, New York, NY 10032 USA; 7 Department of Pharmacology, Columbia University, New York, NY 10032 USA; 8 Excellerate Bioscience Ltd, MediCity, Nottingham NG7 2UH UK

## Introduction

**Fig. 1:**
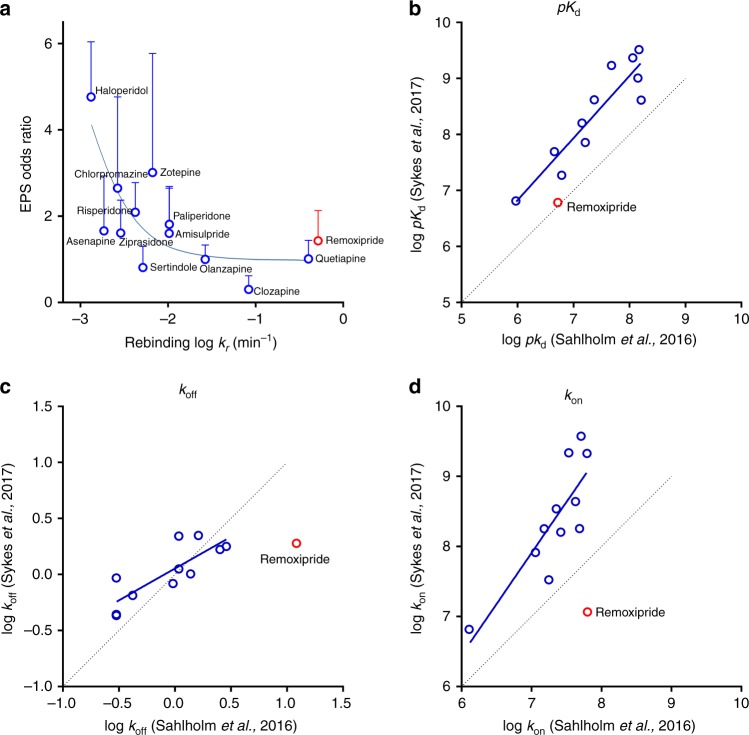
Comparison of kinetic binding data. **a** Correlation of EPS odds ratio with the rebinding reversal rate, reproduced from Sykes et al.^2^ to include the new odds ratio calculated by Zeberg and Sahlholm^1^. **b**–**d** Comparison of **b** p*K*_d_, **c** *k*_off_, and **d** *k*_on_ values at the dopamine D2 receptor calculated using the GIRK channel assay of Sahlholm et al.^4^ and TR-FRET assay of Sykes et al.^2^ It should be noted that the study by Sahlholm et al.^4^ was performed at 20–22 °C whilst the study of Sykes et al.^2^ was performed at 37 °C

We thank Zeberg and Sahlholm^1^ for their correspondence regarding our recent paper proposing that drug rebinding to the dopamine D_2_ receptor (D_2_R) contributes to the extrapyramidal symptoms (EPS) observed with many antipsychotic drugs^2^. We were gratified to note that, like us, the authors obtained a strong correlation between EPS (derived from ref. ^3^) and the association rates they have measured using a distinct method. We are also pleased that the new odds ratio they calculated for remoxipride fits our rebinding hypothesis, strengthening the overall correlation between EPS and *k*_r_ (Fig. 1a). However, they highlight a discrepancy between the association and dissociation rates of remoxipride, which they report as being much more rapid than the values we determined. This reduces the strength of their own correlation between association rate and EPS that, they have argued, weakens our rebinding hypothesis.

Rather than directly measuring binding at the D_2_R, as in our study, Sahlholm and colleagues^4^ employed an indirect functional readout in recombinant oocytes to assess binding kinetics at the D_2_R. Using an electrophysiological approach, they measure the activation of inwardly rectifying potassium channels (GIRK) as a surrogate of agonist binding at the D_2_R. This approach assumes that there is a direct, linear relationship between agonist occupancy at the receptor and GIRK channel activation. This is not, however, commonly observed for receptor signaling events, even for measurements of proximal events such as G protein activation. Instead, operational models of receptor activation describe a hyperbolic relationship between agonist binding and downstream effector activation^5^.

However, despite these differences in assay methodology, the affinity measurements reported for a selection of antipsychotics are surprisingly similar to ours, with a Pearson’s correlation (*r*_s_) of 0.90 (*P* < 0.0001) between the two data sets (Fig. 1b). This close agreement is also observed for the kinetic parameters, with the only clear exception being remoxipride. This is illustrated in Fig. 1c, d, where the Pearson’s correlation (*r*_s_) for *k*_off_ and *k*_on_ is 0.77 (*P* = 0.0033) and 0.59 (*P* = 0.0428) for the whole data set, but rises to 0.82 (*P* = 0.0021) and 0.84 (*P* = 0.0015) when remoxipride is removed from the analysis. It therefore appears that this discrepancy in kinetics for remoxipride may be related to the compound itself, rather than due to differences in assay methodology. In Sahlholm’s study^4^, remoxipride was tested at a 10-fold higher concentration (100 μM) than any of the other ligands, despite having an apparent *K*_d_ similar to that of clozapine. It is possible that at this higher concentration remoxipride could display off-target effects that contribute to the much faster apparent association rate. For example, several other antipsychotics have previously been shown to directly block GIRK channels expressed in xenopus oocytes^6^. If the high concentrations of remoxipride used in the study of Sahlholm and colleagues^4^ were to directly block GIRK channels, it would manifest in the appearance of a more rapid association to the D_2_R. This potential off-target action is supported by a previous study^7^ that directly measured the dissociation of [^3^H]-remoxipride from the D_2_R. They reported an off rate of 3.2 min^−1^, similar to our own value of 1.9 min^−1^, but almost 4-fold different from the value of 12.2 min^−1^ obtained by Sahlholm and colleagues^4^.

In their commentary, Zeberg and Sahlholm have offered alternative explanations for the discrepancy in kinetic data for remoxipride, which we will now address. The first pertains to potential differences in “tracer” kinetics between our two assay systems. They argue that their “tracer” (essentially the activation and inactivation of the GIRK channel) is much more rapid than the dissociation rate of our fluorescent tracer (F-PPHT), and is therefore more appropriate for assessing rapid binding kinetics. Indeed, they require a rapid tracer because they make the assumption that the rate-limiting step is binding and unbinding of the antagonist, rather than the combined summation of the steps between dopamine binding, receptor-mediated activation of G proteins and subsequent GIRK channel activation. This, they have acknowledged, creates an upper limit of sensitivity (or ceiling effect) that limits their analysis to decay rates slower than ~1 s and antagonist concentrations below 10 μM^4^. An additional complication is their assumption that once the antagonist dissociates, dopamine will immediately occupy the empty receptor. This is dependent upon using a concentration of dopamine that is high enough to completely out-compete the antagonist following washout. In a previous study, 100 nM dopamine was found to be insufficient^8^, so in their latest paper^4^ they increase this to 100 μM. It is important to note, however, that even at this high agonist concentration not all antagonists can be competed back to baseline. This observation suggests that 100 μM dopamine is not sufficient to fully out-compete all antagonists, as is assumed in their data analysis, but rather that a new equilibrium is established with partial occupancy of the receptor population by the antagonist. This will slow the overall observed reversal rate.

**Fig. 2:**
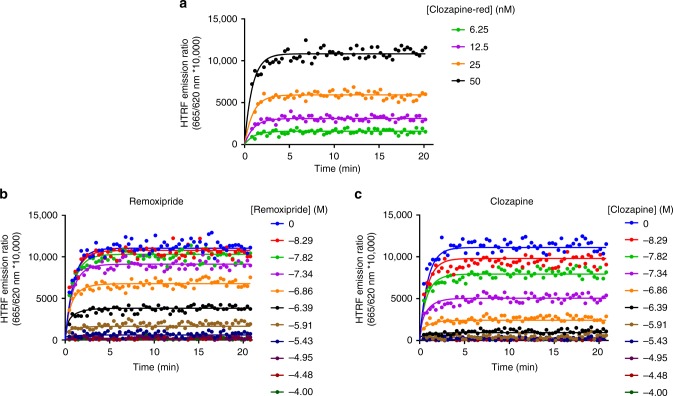
Determination of tracer and unlabelled compound kinetic parameters. **a** Observed association of clozapine-red to the human dopamine D_2L_ receptor. Data presented in singlet from a representation of 4 experiments. Clozapine-red competition association curves in the presence of **b** remoxipride and **c** clozapine. All binding reactions were performed in the presence of GppNHp (100 μM) and non-specific binding levels determined by inclusion of haloperidol (10 μM). Kinetic data were fitted to equations previously described to calculate *k*_on_ and *k*_off_ values for the unlabelled ligands; these are summarized in Table 1. Data are presented as singlet values from a representative of three to four experiments. All data used in these plots are detailed in Table 1

To avoid the above assumptions, we chose to measure binding directly to the D_2_R and use a mathematical approach that explicitly incorporates the association and dissociation rates of the tracer molecule when calculating the kinetics of competing antagonists. In this way we avoid the necessity for the tracer kinetics to be much more rapid than the test compound. As mentioned by Zeberg and Sahlholm, we have previously found that using this assay, rapid competitor dissociation rates cannot be accurately determined using a tracer ligand with a much slower rate of dissociation. This observation was made using the slowly dissociating antagonist spiperone as a tracer (*k*_off_ = 0.026 min^−1^)^2, 9^. For this reason we selected PPHT-red as the tracer ligand for our most recent studies as it has a dissociation rate 10-fold higher than spiperone of 0.52 min^−1^. We acknowledge that there will still be a theoretical limit of detection with this tracer, but believe 1.9 min^−1^ (our *k*_off_ for remoxipride) is within our levels of detection as we have measured ligands with more rapid dissociation rates (e.g., ropinirole at 2.6 min^−1^)^9^. We have also independently verified the binding kinetics of remoxipride and clozapine using another rapidly dissociating tracer, clozapine-red. The association and dissociation values calculated for remoxipride using this tracer were 1.00 × 10^7^ M^−1^ min^−1^ and 1.63 min^−1^, respectively, demonstrating tracer independence with regard to kinetic determinations. Data from these experiments are shown in Fig. 2 and a comparison of kinetic values obtained with the fluorescent tracer clozapine-red and those obtained previously using PPHT-red are detailed in Table 1.

**Table 1:** Comparison of *k*_on_ and *k*_off_ values obtained using the tracers F-PPHT and F-clozapine

	F-PPHT		F-Clozapine	
Compound	*k*_off_ (min^−1^)	*k*_on_ (M^−1^ min^−1^)	*k*_off_ (min^−1^)	*k*_on_ (M^−1^ min^−1^)
Remoxipride	1.90 ± 0.55	1.16 ± 0.37 × 10^7^	1.63 ± 0.16	1.00 ± 0.27 × 10^7^
Clozapine	1.67 ± 0.25	8.23 ± 1.42 × 10^7^	1.78 ± 0.28	4.84 ± 1.02 × 10^7^

F-Clozapine’s association rate was measured at 2.71 × 10^6^ M^−1^ min^−1^ with a *k*_off_ value of 0.79 min^−1^ at 37 °C in HBSS. PPHT-red experiments were performed, as previously described (Sykes et al.^2^)

Data are mean ± SEM from 3 to 4 experiments

Zeberg and Sahlholm also suggest that the inclusion of GppNHp in our assay might influence the observed kinetics for remoxipride, and that their assay is more physiologically relevant because it measures interactions with the higher affinity “functional” state of the receptor. This high affinity (G protein-coupled) state is, however, extremely transient in a whole cell due the high intracellular concentrations of GTP. We include GppNHp with our membrane preparations to mimic this high concentration. All current evidence suggests that once the agonist dissociates, then in a whole cell, the receptor rapidly reverts to the inactive uncoupled state before an antagonist binds. This, as previously discussed, is supported by the good agreement of the kinetic values between our membrane assay and their whole cell oocyte system, with the only clear exception being remoxipride (Fig. 1b–d). In summary, although each method for measuring the kinetics of unlabelled GPCR ligands has its flaws, our direct binding approach at the receptor minimizes issues associated with occupancy-response assumptions and off-target activities.

The final comment raised by Zeberg and Sahlholm relates to the antipsychotic thioridazine, which they state has a favourable EPS profile despite its rapid association rate. Note that this drug was not included in the meta-analysis of Leucht et al. so could not be included in our correlation. As discussed in our paper, it was not our intension to present the rebinding hypothesis as the only mechanism that might predict EPS. Rather, many antipsychotics display complex poly-pharmacology that may also contribute to their relative efficacy and side-effect profiles. For example the superior EPS profile of thioridazine may relate to off-target activity at the muscarinic M_1_ receptor^10^. Our study revealed a compelling correlation between EPS and association rate, and we propose a mechanism of drug-rebinding that can explain this correlation^2^. Given the complex action of antipsychotic drugs it would, however, be naive to expect that all drugs would fall within this correlation. Those that do not may act via distinct mechanisms. Indeed, in our study we observed that aripiprazole, a drug that acts as a D_2_R partial agonist rather than an antagonist, was an outlier. We proposed that this was because of its distinct action at the D_2_R. As such, we feel that one should not discount the potential importance of drug rebinding at the D_2_R due to a small number of drugs that do not fit this correlation. Instead, just as the work of Seeman and colleagues^11^ stimulated our study, we hope that our work will inspire future studies aimed at carefully testing our hypothesis and unraveling the complex mechanisms that determine antipsychotic drug efficacy.

## Methods

### Materials

**Fig. 3:**
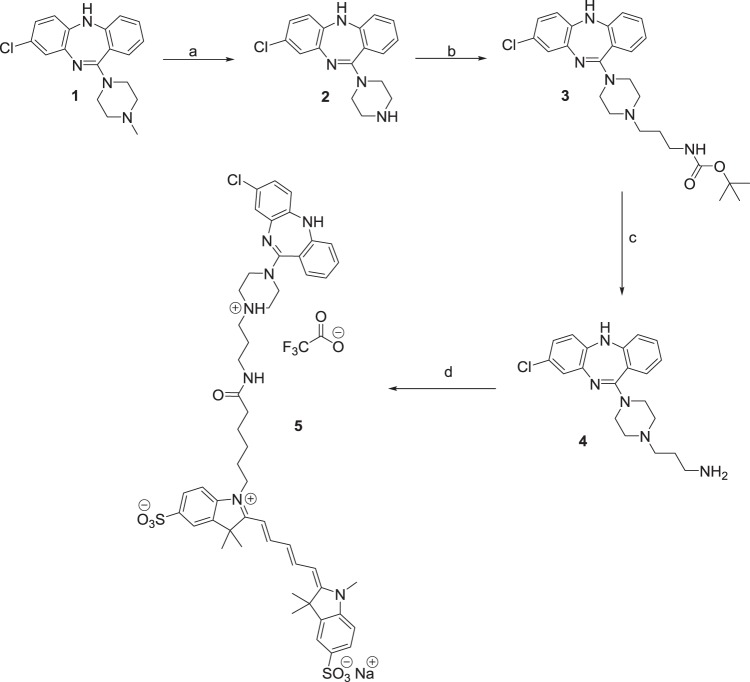
Synthesis of sulfo-Cy5 fluorescently labelled derivative of clozapine. Reagents and conditions: **a** 1-chloroethyl chloroformate, 1,2-DCE, N_2_, MeOH, 0 °C → reflux, 24 h, 54%; **b** *tert*-butyl (3-bromopropyl)carbamate, NaI, DIPEA, N_2_, CH_3_CN, reflux 24 h, 80%; **c** TFA/DCM, RT, 1–2 h, basic workup, 97%; **d** sulfo-Cy5 *N*-hydroxysuccinimidyl (NHS) ester, DMF, RT, 12 h, 18%

Clozapine-red was synthesized in house as follows (Fig. 3).

### Experimental

*N*-Desmethylclozapine (**2**): Clozapine (**1**, 2.50 g, 7.65 mmol) was dissolved in 1,2-dichloroethane (20 mL) under N_2_ and cooled to 0 °C. 1-Chloroethyl chloroformate (3.30 mL, 30.6 mmol) was added dropwise to the reaction mixture. After 10 min, the reaction mixture was warmed up to r.t and subsequently heated at reflux for 24 h. The brown reaction mixture was concentrated in vacuo, and the residue was dissolved in methanol (30 mL) and heated at 50 °C for 2 h, cooled, and again concentrated in vacuo. The resulting residue was partitioned between ethyl acetate (50 mL) and 1 M aqueous hydrochloric acid (50 mL). The aqueous layer was collected and the pH adjusted to ~10 using concentrated sodium hydroxide then extracted with ethyl acetate (3 × 50 mL). The combined organic layers were washed with water (50 mL) and saturated brine (50 mL), dried over anhydrous Na_2_SO_4_, filtered and evaporated to dryness. Purification was achieved via column chromatography (chloroform/methanol, 10%) to give a yellow foam (1.30 g, 54% yield). ^1^H NMR (CDCl_3_) δ 2.57 (br s, 1H), 3.01 (m, 4H), 3.48 (m, 4H), 4.90 (s, 1H), 6.61 (d, *J* 8.3Hz, 1H), 6.81–6.84 (m, 2H), 7.02 (td, *J* 7.6, 1.1Hz, 1H), 7.06 (d, *J* 2.4Hz, 1H), 7.25–7.32 (m, 2H). ^13^C NMR (CDCl_3_) δ 45.7 (CH_2_), 48.2 (CH_2_), 120.1 (CH), 120.2 (CH), 123.2 (CH), 123.2 (CH), 123.5 (C), 126.9 (CH), 129.2 (C), 130.4 (CH), 132.0 (CH), 140.5 (C), 141.9 (C), 152.8 (C), 163.1 (C).

*tert*-Butyl (3-(4-(8-chloro-5H-dibenzo[b,e][1,4]diazepin-11-yl)piperazin-1-yl)propyl)carbamate (**3**): *N*-Desmethylclozapine (**2**, 500 mg, 1.60 mmol), sodium iodide (240 mg, 1.60 mmol) and *N*,*N*-diisopropylethylamine (0.31 mL, 1.76 mmol) were added to acetonitrile (30 mL) under N_2_. *tert*-Butyl (3-bromopropyl)carbamate (419 mg, 1.76 mmol) was added and the reaction mixture was heated at reflux for 24 h. After cooling to room temperature the solvent was removed and the residue was dissolved in ethyl acetate (30 mL), washed with water (2 × 30 mL) followed by brine (50 mL), dried over anhydrous Na_2_SO_4_, filtered and concentrated to give the crude product. Further purification via column chromatography (chloroform/methanol, 5%) gave the title compound as a yellow foam (602 mg, 80%). ^1^H NMR (CDCl_3_) δ 1.44 (s, 9H), 1.69 (m, 2H), 2.46 (t, *J* 6.8Hz, 2H), 2.52 (m, 4H), 3.21 (m, 2H), 3.47 (m, 4H), 4.90 (s, 1H), 5.26 (br s, 1H), 6.61 (d, *J* 8.3Hz, 1H), 6.80–6.83 (m, 2H), 7.01 (td, *J* 7.6, 1.1Hz, 1H), 7.06 (d, *J* 2.4Hz, 1H), 7.24–7.32 (m, 2H). ^13^C NMR (CDCl_3_) δ 26.6 (CH_2_), 28.6 (CH_3_), 39.9 (CH_2_), 47.3 (CH_2_), 53.3 (CH_2_), 57.0 (CH_2_), 79.0 (C), 120.1 (CH), 120.2 (CH), 123.2 (CH), 123.2 (CH), 123.5 (C), 126.9 (CH), 129.2 (C), 130.4 (CH), 132.0 (CH), 140.5 (C), 141.9 (C), 152.8 (C), 156.2 (C), 162.8 (C).

3-(4-(8-Chloro-5H-dibenzo[b,e][1,4]diazepin-11-yl)piperazin-1-yl)propan-1-amine (**4**): Compound **3** (328 mg, 0.698 mmol) was dissolved in DCM (10 mL) and TFA (2 mL) was added dropwise. Stirring at room temperature occurred for 1–2 h before the reaction mixture was diluted with a further 20 mL of DCM. Saturated K_2_CO_3_ (20 mL) was added slowly and the mixture further extracted with 3 × 20 mL portions of DCM. The combined organic layers were further washed with water (50 mL), brine (50 mL), dried over anhydrous Na_2_SO_4_, filtered and concentrated to give the product as a yellow foam which was used in the subsequent reaction without further purification (249 mg, 97%). ^1^H NMR (CDCl_3_) δ 1.30 (br s, 2H), 1.67 (m, 2H), 2.46 (m, 2H), 2.53 (m, 4H), 2.77 (t, *J* 6.8Hz, 2H) 3.47 (m, 4H), 4.90 (s, 1H), 6.61 (d, *J* 8.3Hz, 1H), 6.80–6.83 (m, 2H), 7.01 (td, *J* 7.6, 1.1Hz, 1H), 7.06 (d, *J* 2.4Hz, 1H), 7.24–7.32 (m, 2H). ^13^C NMR (CDCl_3_) δ 30.7 (CH_2_), 40.9 (CH_2_), 47.4 (CH_2_), 53.4 (CH_2_), 56.6 (CH_2_), 120.1 (CH), 120.2 (CH), 123.2 (CH), 123.2 (CH), 123.6 (C), 126.9 (CH), 129.2 (C), 130.4 (CH), 132.0 (CH), 140.5 (C), 141.9 (C), 152.8 (C), 162.9 (C).

1-(6-((3-(4-(8-Chloro-5H-dibenzo[b,e][1,4]diazepin-11-yl)piperazin-1-ium-1-yl)propyl)amino)-6-oxohexyl)-3,3-dimethyl-2-((1E,3E,5E)-5-(1,3,3-trimethyl-5-sulfoindolin-2-ylidene)penta-1,3-dien-1-yl)-3H-indol-1-ium-5-sulfonate 2,2,2-trifluoroacetate (**5**): The free amine (**4**, 1 equiv.) followed the sulfo-Cy5 NHS ester (1 equiv.) was added to DMF (1 mL) under a N_2_ atmosphere. The reaction was stirred at room temperature for 12 h in the absence of light, then purified immediately via preparative-HPLC. The clean fractions were collected, pooled and the solvent removed via lyophillization to obtain the product as the TFA salt. Blue solid (2 mg, 18%). ^1^H NMR (*d*_6_-DMSO) δ 0.83 (m, 2H), 1.15–1.29 (m, 5H), 1.51 (m, 2H), 1.69 (s, 12H), 2.02 (t, *J* 7.1Hz, 2H), 2.97 (m, 2H), 2.97–3.40 (m, 7H, under water peak), 3.60 (s, 3H), 3.94 (m, 2H), 4.12 (m, 2H), 6.29 (m, 2H), 6.56 (t, *J* 12.6Hz, 1H), 6.88–6.94 (m, 3H), 7.01–7.09 (m, 2H), 7.29–7.41 (m, 5H), 7.64–7.66 (m, 2H), 7.83–7.84 (m, 3H), 8.36 (m, 2H), 9.58 (br s, 1H). HPLC purity (*λ* = 214 nm): 95%, *t*_R_ = 4.89 min. HRMS (ESI)-TOF (*m/z)*: [M + H]^+^ 994.3762 calcd for C_52_H_60_ClN_7_O_7_S_2_; found [M + H]^+^ 994.3765.

All other reagents and materials were obtained as previously described (Sykes et al.^2^).

Culture and terbium labeling of SNAP-tagged D_2L_ cells plus membrane preparation: CHO-D_2L_ cells were cultured and membranes prepared, as previously described^2^.

Fluorescent ligand-binding assays: Experiments using clozapine-red were performed in white 384 well Optiplate plates, in assay binding buffer, HBSS containing 20 mM HEPES and 0.02% pluronic acid pH 7.4, 100 μM GppNHp and 0.1% ascorbic acid. Haloperidol (10 μM) was used to define the level of nonspecific binding.

Determination of clozapine-red binding kinetics: The association rate (*k*_on_) and dissociation rate (*k*_off_) values of clozapine-red was determined using multiple different concentrations of clozapine-red. Clozapine-red (50–6.25 nM) was incubated with human D_2L_ CHO cell membranes (2 μg/well) in assay binding buffer (final assay volume, 40 μl). Specific binding of clozapine-red bound to the D_2L_ receptor was measured at 20 s intervals by HTRF detection allowing construction of clozapine-red association curves. Data were globally fitted to the association kinetic model to derive a single best-fit estimate for *k*_on_ and *k*_off_.

Competition binding kinetics: To determine the association and dissociation rates of D_2_R ligands, we used a competition kinetic binding assay. This methodology involves the simultaneous addition of both a fluorescent ligand (the tracer) and unlabelled competitor to the receptor preparation of interest in this case the human dopamine D_2L_R, so that at *t* = 0 all receptors are unoccupied. 50 nM clozapine-red (a concentration which avoids ligand depletion in this assay volume), was added simultaneously with the unlabelled compound (at *t* = 0) to CHO cell membranes containing the human dopamine D_2L_R (2 μg per well) in 40 μl of assay buffer. The degree of clozapine-red bound to the receptor was measured at 20 s intervals by HTRF detection.

As described previously nonspecific binding was determined in the presence of haloperidol (10 μM) and was subtracted from each time point. Time points were performed on the same 384 well Optiplate plate maintained at constant temperature, 37 °C with orbital mixing (1 s of 100 RPM/cycle). For determination of rate parameters multiple concentrations of unlabelled competitor were employed and data were globally fitted to simultaneously calculate *k*_on_ and *k*_off_ as previously described.

Signal detection and data analysis: Signal detection was performed on a Pherastar FS (BMG Labtech, Offenburg, Germany) using standard HTRF settings and experiments were analyzed by non-regression using Prism 6.0 (GraphPad Software, San Diego, USA) all as previously described (Sykes et al.^2^).

## Data Availability

The data that support the findings of this study are available from the corresponding author upon reasonable request.
